# Reduction of pruritus and depression using longitudinal patient-reported outcome measures in hemodialysis: a quality improvement project

**DOI:** 10.1186/s41687-026-01003-6

**Published:** 2026-01-21

**Authors:** Luis Naar, Sebastian Mussnig, Janosch Niknam, Florian Brosch, Simon Krenn, Christopher C. Mayer, Joachim Beige, Manfred Hecking

**Affiliations:** 1 Division for Nephrology and Dialysis, Department of Medicine III, Medical University of Vienna, Vienna, Austria; 2 Center for Public Health, Department of Epidemiology, Medical University of Vienna, Vienna, Austria; 3 AIT Austrian Institute of Technology, Center for Health & Bioresources, Vienna, Austria; 4 Kuratorium for Dialysis and Transplantation e.V. (KfH), Neu-Isenburg, Germany; 5 Medical Clinic 2, Martin Luther University Halle-Wittenberg, Halle, Germany

**Keywords:** Hemodialysis, Chronic kidney diseases-associated pruritus (CKD-aP), Depression, Patient-reported outcome measures, Quality improvement, Difelikefalin, Symptom management

## Abstract

**Importance:**

Chronic kidney disease–associated pruritus (CKD-aP) and depression are common but often underrecognized and undertreated in hemodialysis (HD) patients, lowering quality of life and worsening outcomes.

**Objective:**

To evaluate whether the systematic, repeated collection of patient-reported outcome measures (PROMs) integrated into a Plan-Do-Act-Study (PDAS) framework could enhance recognition of CKD-aP and depression and guide treatment.

**Design, setting, and participants:**

127 adult patients receiving thrice-weekly in-center HD were included. PROMs were captured electronically at predefined phases.

**Interventions:**

During Adjustment phases, individualized symptom reports were reviewed by physicians, prompting initiation of difelikefalin for CKD-aP or referral to psychiatric care for depressive symptoms.

**Main outcomes and measures:**

Changes in itch (Worst-Itch Numerical Rating Scale, WI-NRS) and depression (Patient Health Questionnaire-9, PHQ-9) and concordance between patients and nurses.

**Results:**

At baseline, 32% of patients versus 9% of nurses reported pruritus (κ = 0.18) and 27% versus 6% reported depression (κ = 0.19). Following PROM-guided interventions, 16 patients initiated difelikefalin (median WI-NRS reduction of 3 points, *p* < 0.001) and five were referred for psychiatric care (median PHQ-9 reduction of 4.5 points, *p* = 0.015). Patient–nurse agreement improved to κ = 0.66 for pruritus and κ = 0.65 for depression at study end. Ratings of assessment adequacy rose from 38% to 67% among patients and from 55% to 75% among nurses.

**Conclusions and relevance:**

Routine, repeated PROMs markedly improved recognition of CKD-aP and depression, enabled targeted therapy with meaningful relief, and strengthened patient-staff alignment. This PDAS-based strategy supports KDIGO guidance and merits broader adoption to advance patient-centered dialysis care.

**Supplementary Information:**

The online version contains supplementary material available at 10.1186/s41687-026-01003-6.

## Introduction

Patients undergoing hemodialysis (HD) are routinely monitored by nephrologists and health-care staff [1]. Nevertheless, highly prevalent, distressing and potentially modifiable patient-reported symptoms such as chronic kidney disease-associated pruritus (CKD-aP) and depression remain under-assessed and under-documented [2–7].

In the Dialysis Outcomes and Practice Patterns Study (DOPPS) I and II (1996–2004), 60% of HD patients reported pruritus, with 20–40% experiencing moderate to severe symptoms [8, 9]. The prevalence decreased to 37% in DOPPS V (2012-15) data [5] and was reported at 50% among European dialysis patients in 2023, where 60% of those with severe symptoms remained untreated [10].

Failure to manage pruritus effectively not only reduced patients’ quality of life (QoL) through physical discomfort and sleep disruption but could also contributed to increased depression, anxiety and healthcare utilization [11–15]. Antihistamines proved ineffective for the treatment of CKD-aP and are not recommended [12, 16]. Gabapentinoids showed effectiveness against CKD-aP but due to their off-label status and central nervous system-related action (e.g., somnolence, dizziness) they are currently not recommended as first-line treatment for CKD-aP [16]. Difelikefalin, a peripherally acting kappa-opioid receptor agonist reduced itch with minimal central opioid-related adverse effects and is the only widely approved therapy for the treatment for CKD-aP in patients undergoing HD at present [1, 2, 17–19]. Moreover, trials have demonstrated that difelikefalin yields improvements in patient-reported QoL [19, 20],

CKD-aP is associated with depression and depression can lead to pruritus [6]. Depression affects roughly 20–30% of HD patients [6, 21, 22] and is associated with adverse outcomes, including lower adherence to dialysis treatment and dietary restrictions, higher hospitalization rates, reduced QoL and increased mortality risk [23, 24]. Disease-related biological contributors (e.g., uremic toxins, systemic inflammation) and psychological stressors (e.g., anxiety about prognosis and loss of independence) often compound the burden of depression [7, 22]. Social determinants, such as limited family support or lower socioeconomic status, can further exacerbate depressive symptoms [22]. For depression, in HD patients, multidisciplinary strategies, including cognitive-behavioral therapy, selective serotonin reuptake inhibitors (SSRIs), exercise, and psychosocial support, have been shown to improve both clinical and patient-reported outcomes [24–27].

Current management of both, CKD-aP and depression present gaps in the care of HD patients, that could be addressed by repeated, systematic collection of patient-reported outcomes measures (PROMs) [1]. The Kidney Disease Improving Global Outcomes (KDIGO) 2024 Clinical Practice Guideline underscored the importance of routinely evaluating and managing symptoms that significantly affect QoL [1, 18].

The present analysis of a quality improvement (QI) project aimed to evaluate whether repeated use of PROMs for CKD-aP and depression could enhance disease awareness among HD patients and health care staff. Furthermore, we aimed to investigate whether longitudinal PROMS-based therapeutic adjustment had effects on CKD-aP and depression over time.

## Materials and methods

### Population and ethics

In May 2024, the Kuratorium for Dialysis and Transplantation e.V. (KfH, Neu-Isenburg, Germany) implemented a QI project at the KfH dialysis center Weiden (Weiden in der Oberpfalz, Germany) in cooperation with the Medical University of Vienna (Vienna, Austria). The aim of this project was to longitudinally evaluate CKD-aP, depression and fluid status in patients undergoing hemodialysis using objective measures and PROMs, and to adjust treatment if any measures suggested the need thereof. Results from improvement in fluid status are published elsewhere [28]. All adult patients receiving thrice-weekly in-center hemodialysis at the KfH dialysis center Weiden were eligible for participation in the project. No additional inclusion or exclusion criteria were applied beyond regular treatment at the center. Patients were informed about the aims and procedures of the project during routine dialysis sessions and were given the opportunity to decline participation in the PROM assessments. The QI project was authorized by the Institutional Review Board of the KfH with a requirement to process pseudonymized data internally. The objectives of the project were defined a priori as part of a prospectively planned QI initiative. The current manuscript presents a retrospective analysis of the data collected during this QI project for the purpose of scientific dissemination. Prior to the retrospective analysis and publication of the project data, a full protocol with all additional documents was submitted for approval to the Ethics Committee of the Bavarian State Medical Association. The applicant (MH) was informed in writing that an ethics vote through the board of the ethics committee was not formally required for data analysis and publication, as previously reported in the publication of fluid data from this project [29]. The methodology used within the project was compatible with routine clinical practice, did not pose additional risks to patients and was therefore conducted without formal written informed consent, as previously reported to the aforementioned ethics committee The reporting of this quality improvement project was guided by the SQUIRE 2.0 (Standards for Quality Improvement Reporting Excellence) recommendations.

### Design and longitudinal assessment

**Fig. 1:**
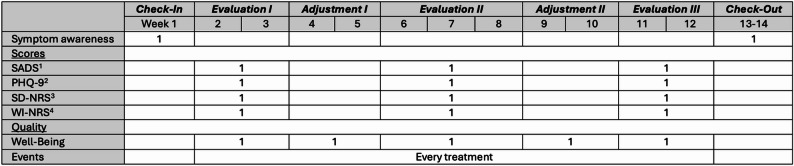
Flowchart of the QI project. The QI project lasted 14 weeks. Patients and nurses reported how they perceived the patient’s pruritus and depression status at *Check-In* and *Check-Out*. Patient-reported outcome measures (PROMs) were captured at least once each phase between *Evaluation I* to *Evaluation III*. Data were summarized before each *Adjustment* phase. Subsequent treatment adjustments were guided by these summaries. Abbreviations: (1) SADS: Self-Assessed Disease Severity scale for pruritus; (2) PHQ-9: Patient Health Questionnaire for depressive symptoms; (3) SD-NRS: Sleep-Disturbance Numerical Rating Scale; (4) WI-NRS: Worst-Itch Numerical Rating Scale

This QI project was conceptualized in alignment with the Plan-Do-Act-Study (PDAS) principle. It was structured into a *Check-In* phase, alternating periods of *Evaluation* and *Adjustment* phases, and a final *Check-Out* phase (Fig. 1). The project lasted for 14 weeks. During the first week (*Check-In*), patients were introduced to the project’s purpose and underwent initial self-assessments for pruritus and depression (Supplement Table S1). At the same time, nurses provided their clinical judgments regarding each patient’s pruritus and depression status (Supplement Table S2). Subsequent assessments took place in weeks 2–3 (*Evaluation I*), 6–8 (*Evaluation II*), and 11–12 (*Evaluation III*), using electronically captured PROMs. Patients and nurses accessed the surveys (Typeform S.L., Barcelona, Spain) either directly through a tablet computer available at the HD facility or through the Mizu smartphone application (Carealytix Digital Health GmbH, Hohendilching, Germany) that had to be downloaded and set up on the patients’ personal devices. During weeks 4–5 (*Adjustment I*) and 9–10 (*Adjustment II*), all collected PROM data were exported and compiled into individualized summary reports that visualized each patient’s scores and thresholds. These reports were made available to the attending nephrologists prior to routine dialysis rounds. Physicians reviewed the PROM summaries together with each patient during routine encounters and decided whether treatment adjustments were indicated. Decisions to initiate difelikefalin or to refer a patient for psychiatric care were documented in the patient’s medical record as part of routine care. No automated electronic decision support systems were implemented. Rather, PROM reports served as structured decision aids to support clinical judgement. PROM reports were not directly integrated into the electronic health record but were stored on a secure internal server and reviewed alongside the chart. In weeks 13–14 (*Check-Out*), both patients and nurses were surveyed again to evaluate their perceptions regarding pruritus and depression following the implemented interventions (Supplement Table S5-S6).

### Patient-reporting outcome measures

PROMs were selected based on their established use in prior studies addressing CKD-aP and depression, and their feasibility for routine implementation in a QI setting. We used the Worst-Itch Numerical Rating Scale (WI-NRS) for itch intensity (0 = no itch, 10 = worst imaginable itch), the Self-Assessed Disease Severity (SADS) scale for impairment of QoL due to itching (A = mild, B = moderate, C = severe), the Sleep Disturbance Numerical Rating Scale (SD-NRS) for sleep problems (0 = no disturbance, 10 = extreme disturbance), a self-reported well-being scale (0 = very good, 4 = bad), and the Patient Health Questionnaire (PHQ-9) for depressive symptoms (0 = no depressive symptoms, 27 = severe depressive symptoms) (Supplement Table S3–S4). WI-NRS, SADS and SD-NRS are validated instruments for the assessment of CKD-aP and related domains and have demonstrated good reliability and responsiveness to change in patients with CKD-aP, while the PHQ-9 is a widely used, validated instrument for depressive symptoms in medical populations, including patients with CKD [19, 25, 30]. In line with previous work, we defined CKD-aP as WI-NRS ≥ 4, corresponding to at least moderate itch intensity, and considered depressive symptoms present at PHQ-9 ≥ 5, capturing at least mild depressive symptom burden in this QI context. The single-item well-being question was developed pragmatically for this project and has not undergone formal psychometric validation.

### Statistical analysis

Continuous variables were summarized as mean and standard deviation [Q1; Q3], and nominal or ordinal variables as counts (percentages). CKD-aP was defined as WI-NRS score ≥ 4 and depression was classified as present if PHQ-9 score ≥ 5. Agreement between subjective awareness of pruritus (patient and nurse reports of “no pruritus” vs. “pruritus”) and depression (patient and nurse reports of “no depression” vs. “depression”) was assessed via contingency tables at *Check-In* and *Check-Out*. The interrater (patient vs. nurse) reliability was analyzed by Cohen’s kappa. Linear bar charts were used for the descriptive visualization of individual scores over time, presented as means with 95% confidence intervals (CI 95%). To evaluate changes in PROMs over time in response to the intervention ([a] difelikefalin and [b] psychiatric referral), the Friedman test was applied for within-subject comparisons across repeated measurements. In the case of a statistically significant overall effect (α = 0.05), post-hoc pairwise comparisons were conducted using the Wilcoxon signed-rank test, with Bonferroni correction. All statistical analyses were based on complete case data for each respective time point. No imputation of missing values was performed. To control for potential confounding effects all cases were systematically examined for overlap between initiation of difelikefalin treatment and referral for psychiatric care. All statistical analyses and visualizations were conducted using R (version 4.4.1) and RStudio for macOS.

## Results

### Study population

In May 2024, a total of 133 patients received dialysis at the KfH center in Weiden, Germany. Of these, 127 patients provided data for this QI project between May 27 and September 14. Six patients treated at the center during the project period did not provide PROM data, mainly due to logistical reasons (e.g., hospitalization, transfer) and personal preference. The cohort comprised 88 men (69%) and 39 women (31%). The median age was 69 years [61; 82] and the median dialysis vintage was 39 months [18; 68]. The most common causes of kidney failure were glomerular disease (45 patients [40%]) and diabetic nephropathy (25 patients [22%]). A medical record of depression was evident for 8 patients (6%) and pruritus was documented in one patient. At baseline, difelikefalin was used by 6 patients (5%) and antidepressants were used by 5 patients (4%) (Table 1).

**Table 1:** Baseline patient characteristics

Variable	*N*	Overall
Age, years	127	69 (14)
Sex	127	
Female		39 (31%)
Male		88 (69%)
Dialysis modality	119	
Hemodiafiltration		6 (5.0%)
Hemodialysis		113 (95%)
Dialysis vintage, months	126	56 (61)
Residual diuresis, mL	72	636 (552)
Cause of kidney failure	113	
Diabetic		25 (22%)
Glomerular		45 (40%)
Other		16 (14%)
Vascular		27 (24%)
History of kidney transplant	127	6 (4.7%)
Type 2 diabetes mellitus	127	47 (37%)
Heart failure (HFrEF or HFpEF)	127	36 (28%)
Arterial hypertension	127	102 (80%)
Coronary artery disease	127	51 (40%)
History of stroke	127	16 (13%)
History of depression	127	8 (6%)
Difelikefalin at baseline	127	1 (0.1%)
Difelikefalin at baseline	127	6 (5%)
Antidepressant at baseline	127	5 (4%)

The data are reported as frequency (percentage) or mean (standard deviation). Abbreviations: HFrEF: heart failure with reduced ejection fraction; HFpEF: heart failure with preserved ejection fraction

### *Check-In*: Self-reported CKD-aP and depression

**Fig. 2:**
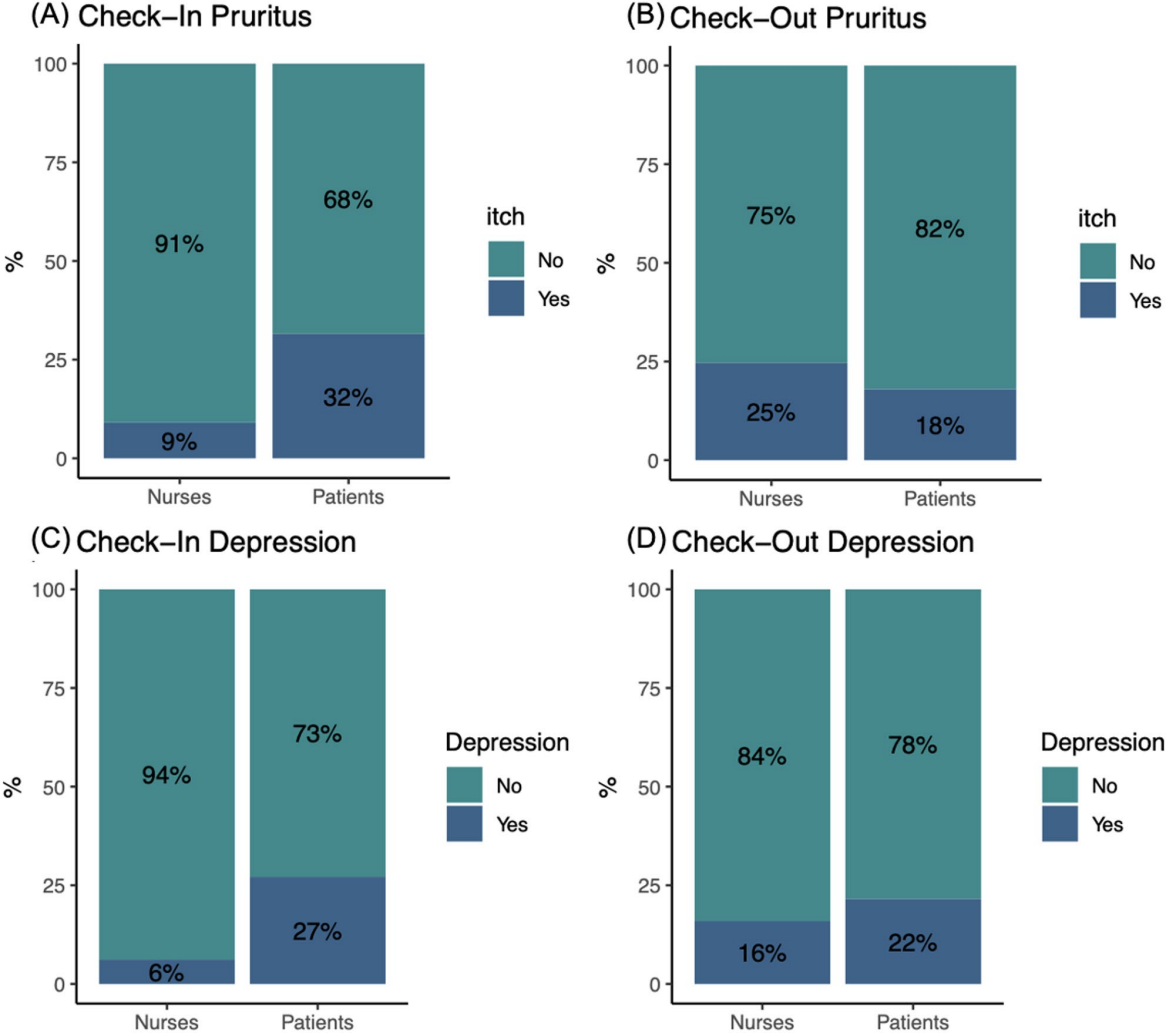
Prevalence of pruritus and depression at *Check-In* and *Check-Out.* The upper panels display the prevalence of pruritus at *Check-In* and *Check-Out* as assessed by nurses (left) and patients (right). The lower panels display the prevalence of depression at *Check-In* and *Check-Out* as assessed by nurses (left) and patients (right)

At *Check-In*, 42 patients (32%) self-reported experiencing “pruritus”, while nurses assessed that only 12 patients (9%) had pruritus. The agreement between nurse and patient evaluations of pruritus was low (Kappa = 0.18, Fig. 2). For depression, 34 patients (27%) reported feeling depressed, while nurses assessed that 7 patients (6%) had depression. Nurse–patient agreement on depression was low (Cohen’s Kappa = 0.19, Fig. 2).

### Evaluation phases and adjustment phases

**Fig. 3:**
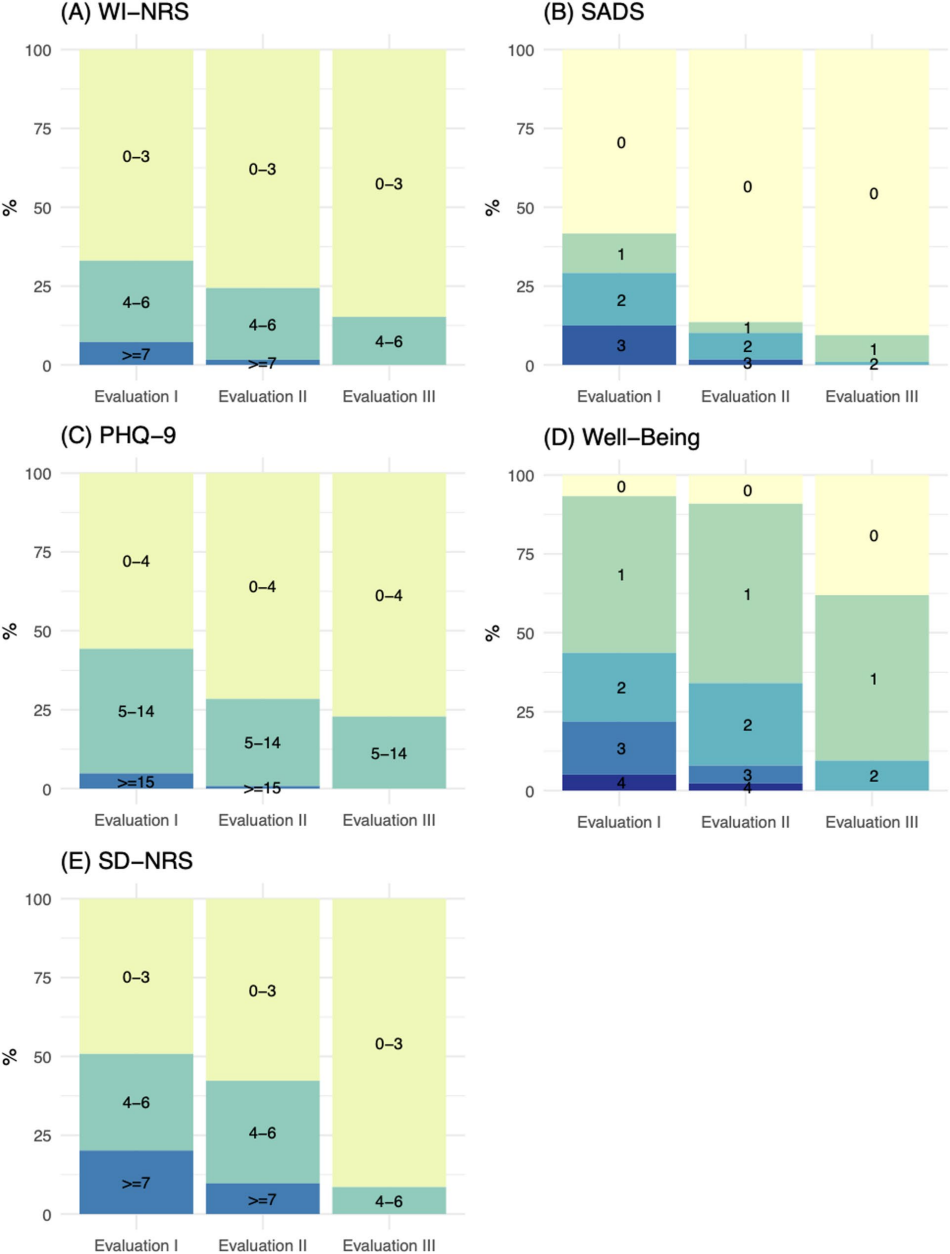
Qualitative variables throughout the QI project. This figure presents the distribution of qualitative variables across different phases of the Quality Improvement (QI) project. The x-axis represents the different project phases: *Evaluation I*,* Evaluation II*, *Evaluation III*. The y-axis represents the percentage (%) of patients within each category

During the first evaluation, 36 patients (29%) reported a WI-NRS score ≥ 4. Of these, 3 were already receiving difelikefalin and 15 were newly initiated on difelikefalin, at their physician’s discretion. Based on PHQ-9 scores, 53 patients (30%) were classified as having mild depressive symptoms, 12 (9%) moderate, 5 (4%) moderately severe, and 1 (0.1%) was classified as having severe depression. At their physicians’ discretion, 5 patients, including the patient with severe symptoms, were referred for psychiatric care. In the second adjustment phase one additional patient was newly started on difelikefalin and no additional patient was referred to psychiatric care (Fig. 3). No patient received both difelikefalin and a psychiatric referral during the project period. Thus, the two intervention groups were mutually exclusive.

### Changes in PROMs in patients who received difelikefalin

**Fig. 4:**
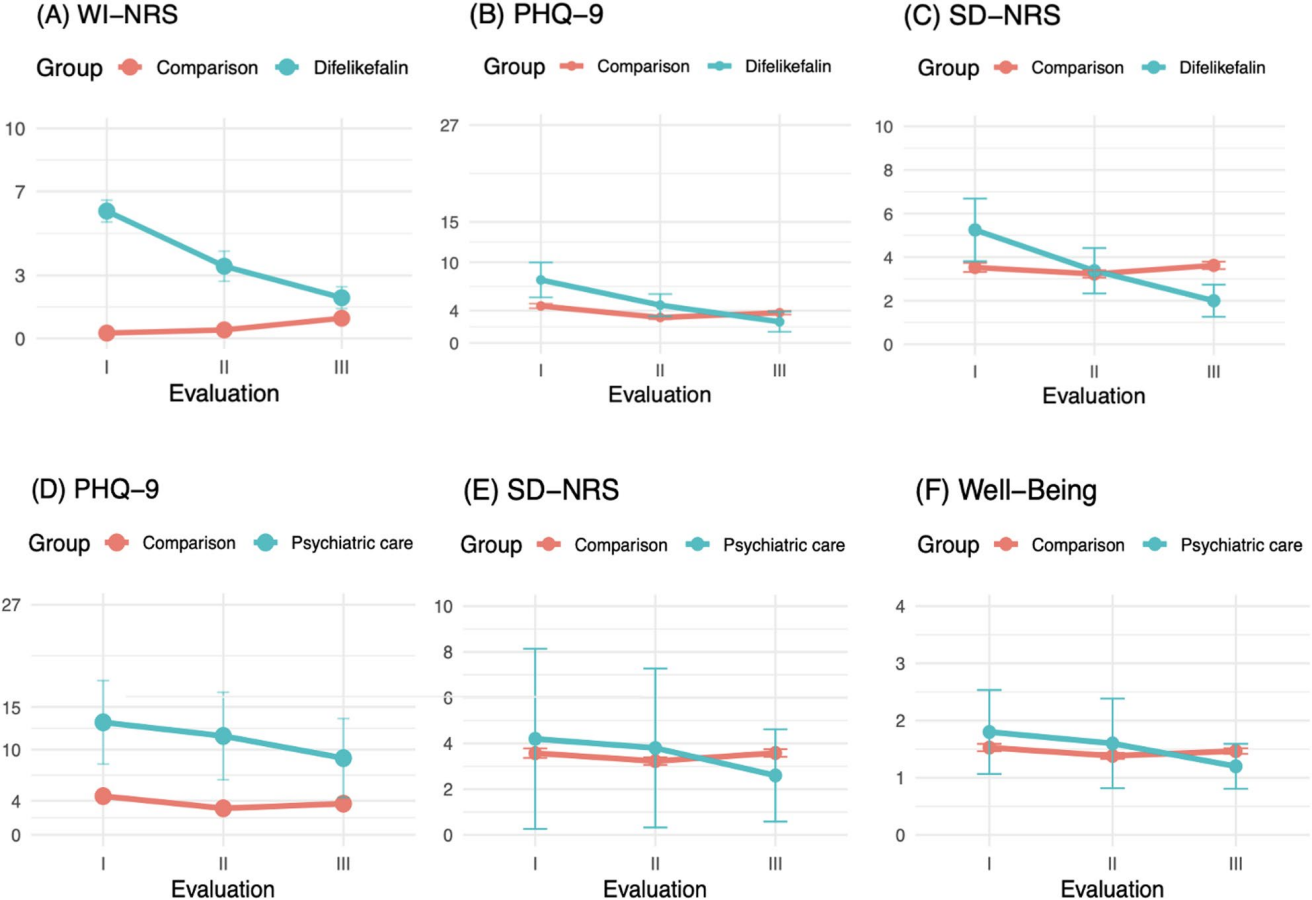
Intervention groups over QI project. This figure illustrates the progression of PROMs over the phases of the quality improvement (QI) project. Panels **A**-**C** show patients treated with difelikefalin (turquois) and those who did not receive difelikefalin (red). Panels **D**-**F** shown patients who were referred to psychiatric care (turquois) and those who did not receive a referral (red). The horizontal axis represents the different phases of the QI project, including evaluation and adjustment periods. The vertical axis represents the respective scores. Abbreviations: PHQ-9: Patient Health Questionnaire for depressive symptoms; SD-NRS: Sleep-Disturbance Numerical Rating Scale; WI-NRS: Worst-Itch Numerical Rating Scale

Significant changes over time were observed for WI-NRS scores in patients receiving difelikefalin (*N* = 16; Friedman χ² (2) = 28.8, *p* < 0.001). Post-hoc Wilcoxon signed-rank tests with Bonferroni correction showed significant reductions between all evaluation phases (*Evaluation I* vs. *II*: adjusted *p* = 0.002; *Evaluation I* vs. *III*: adjusted *p* = 0.001; *Evaluation II* vs. *III*: adjusted *p* = 0.005), corresponding to an overall median reduction of 3 points in WI-NRS over the course of the project. Similarly, significant longitudinal changes were found for PHQ-9 (Friedman χ² (2) = 22.3, *p* < 0.001), SADS (χ² (2) = 22.8, *p* < 0.001), and SD-NRS (χ² (2) = 12.9, *p* = 0.002; Fig. 4). For PHQ-9, all pairwise comparisons remained significant after Bonferroni correction (*Evaluation I* vs. *II*: adjusted *p* = 0.016; *Evaluation I* vs. *III*: adjusted *p* = 0.002; *Evaluation II* vs. *III*: adjusted *p* = 0.011), as did all comparisons for SADS (adjusted *p* = 0.008, 0.002, and 0.044, respectively). For SD-NRS, significant improvements were observed between *Evaluation I* and *III* (adjusted *p* = 0.007) and between *Evaluation II* and *III* (adjusted *p* = 0.036), but not between *Evaluation I* and *II* (adjusted *p* = 0.074). Changes in subjective well-being did not reach statistical significance (*p* = 0.072).

In contrast, among patients who did not receive difelikefalin, WI-NRS scores did not show a meaningful improvement over time and were, on average, slightly higher at the end of the project than at *Evaluation I*, while PHQ-9 scores in these non-treated patients remained relatively stable across project phases.

### Changes in PROMs in patients who were referred to a psychiatric specialist

Among patients who were referred to a psychiatric specialist (*N* = 5), a significant overall change over time was observed for PHQ-9 scores (Friedman χ² (2) = 8.4, *p* = 0.015). Post-hoc Wilcoxon signed-rank tests with Bonferroni correction showed significant reductions between *Evaluation I* and *III* (*Evaluation I* vs. *II*: adjusted *p* = 0.174; *Evaluation I* vs. *III*: adjusted *p* = 0.015; *Evaluation II* vs. *III*: adjusted *p* = 0.174), corresponding to an overall median reduction of 4.5 points in PHQ-9 over the course of the project. In contrast, among patients who were not referred to psychiatric care, PHQ-9 scores remained relatively stable across project phases.

For the same patients referred to psychiatric care, Friedman tests for WI-NRS, SADS and SD-NRS did not indicate significant longitudinal changes.

### *Check-Out*: Self-reported CKD-aP and depression

At *Check-Out*, 22 patients (18%) subjectively reported pruritus, while nurses assessed that 31 patients (25%) had pruritus. Nurse–patient agreement for pruritus was strong (Cohen’s Kappa = 0.66). Regarding depression, 28 (22%) patients self-reported feeling depressed, while nurses assessed that 20 patients (16%) had depression. Nurse–patient agreement was strong (Cohen’s Kappa = 0.65; Fig. 2).

### Adequacy of the assessment

The assessment of pruritus was considered to be adequate by 55% of nurses and 38% of patients at *Check-In*, rising to 75% and 67% at *Check-Out*, respectively. For depression, this proportion increased from 42% of nurses and 31% of patients at *Check-In*, to 58% and 57% at *Check-Out*.

## Discussion

In this single-center QI project, we demonstrated that the systematic and repeated use of PROMs can enhance the recognition and management of both CKD-aP and depression in HD patients. This finding is consistent with the patient-centered care paradigm emphasised in the 2024 KDIGO Clinical Practice Guideline, which explicitly recommended routine PROM collection for burdensome symptoms such as itch and mood disorders. Our iterative approach, including pre-defined evaluation and adjustment phases within a PDAS framework, provided continuous feedback that informed targeted therapeutic interventions.

### Enhanced symptom recognition through iterative evaluations

One central observation was the distinct discrepancy of subjective symptom awareness between patients and nurses for pruritus and depressive symptoms at *Check-In*. Our cohort showed a lower prevalence in CKD-aP than the CENSUS-EU cohort, which found CKD-aP in more than 50% of HD patients [11]. Yet both datasets highlight an alarming treatment gap, with only < 18% of symptomatic individuals receiving antipruritic therapy [10]. Regarding depression, nurses considered only 6% of patients to suffer from depression, while 27% of patients reported depressive symptoms. This under-recognition echoes results from the EMPATHY study, where 29% of HD patients screened positive for mood disorders but mental-health notes appeared in just 6% of charts [31]. Comparable recognition gaps have been confirmed in a recent analysis, in which nephrologists missed ≥ 1 moderate-to-severe symptom in 57% of encounters [32]. In our QI project, improved nurse-patient agreement was observed after repeated assessment alongside increase in subjective reports of assessment adequacy in both groups, suggesting that continuous feedback may sensitize healthcare staff to patient-experienced symptoms that might otherwise be underestimated during routine encounters.

### PROMs-driven symptom management yielded clinical improvements

The structured evaluation phases (*Evaluation I–III*) captured dynamic changes in symptom severity over time. Initiation of difelikefalin in almost 60% of patients with WI-NRS ≥ 4 led to a median 3-point reduction in itch intensity in the treated patients, which was similar to the 3- to 4-point improvements reported in the KALM-1/-2 trials [19, 20]. Beyond a decrease in itch severity, we found parallel improvements in PHQ-9, SADS and SD-NRS. CENSUS-EU showed a stepwise rise in depression scores with increasing itch severity [10]. The observed decrease in PHQ-9 after commencement of antipruritic medication in our project therefore reinforces the causal chain, linking pruritus relief to improved depressive symptoms. Five patients were referred to psychiatric care after PROM review. To our knowledge, this is the first study that coupled repeated PROM collection with a formal depression measure in HD, and we showed an improvement in PHQ-9 scores, accompanied by positive trends in sleep quality and overall well-being. Our findings support the notion that PROM-triggered mental-health interventions may be effective and treatment gaps can be resolved within routine dialysis workflows.

### Implementation considerations

Finding the Right Frequency to Sustain Participation. A major strength of this project was its pragmatic integration into routine dialysis workflows using digital platforms. The iterative evaluation and adjustment phases not only facilitated timely therapeutic interventions but also fostered increased trust and communication between patients and clinical staff. This was reflected in the improved agreement between nurses and patients regarding pruritus and depression, as well as increased perceived adequacy of symptom assessment. Nevertheless, the results sparked speculation in our group that “overly” frequent symptom assessment might provoke increased reports of fatigue, tentatively supported by increased WI-NRS scores of non-treated patients over time. In lack of a comparator group, this question remains unanswered. Future interventions may profit from prudently using less burdensome schedules (e.g., monthly PROMs). Importantly, respondent burden in routine dialysis care is driven not only by the frequency of PROM collection but also by the breadth of domains and the total number of items administered [33]. HD patients frequently experience additional distressing symptoms such as pain, fatigue, sleep disturbance, and anxiety, and expanding PROM coverage to these domains could quickly increase the cumulative assessment load [2, 3, 6]. To mitigate this, future interventions may use tailored PROM schedules in which both the frequency and content of questionnaires are adapted to individual symptom trajectories, risk profiles, and patient preferences [18]. Furthermore, computer adaptive testing (CAT)-based PROM systems may offer an efficient way to monitor multiple domains with minimal respondent burden, by dynamically selecting only the most informative items for each patient while maintaining measurement precision [34, 35]. Integrating CAT-based PROMs into PDAS cycles could therefore support broader yet pragmatic symptom surveillance in dialysis populations.

### Limitations and future directions

Several limitations must be acknowledged. The observational design and the relatively short project duration of only 14 weeks limited our ability to assess the long-term sustainability and broader generalizability of these interventions. Although this project was conducted in a single German dialysis center with access to tablet and smartphone-based PROM collection, the underlying PDAS framework is not inherently dependent on sophisticated digital infrastructure. The key components, systematic PROM capture, structured feedback cycles, and predefined adjustment phases, could be implemented in centers with different staffing ratios using paper-based instruments or simple summary reports. In dialysis units with fewer staff per patient, less frequent PROM assessments or targeted follow-up of high-risk patients may be required to maintain feasibility while preserving the benefits of symptom monitoring. In particular, only five patients were referred for psychiatric care, and we did not systematically collect detailed information about the type and intensity of psychiatric interventions received (e.g., psychotherapy, pharmacotherapy strategy’s, combined approaches). Consequently, our ability to interpret the observed changes in PHQ-9 scores and to attribute them to specific mental-health treatments is limited, and these findings should be viewed as exploratory. Additionally, the limited number of patients receiving specific treatments more broadly hindered more detailed subgroup analyses. We did not systematically record reasons why some patients with WI-NRS ≥ 4 did not initiate difelikefalin, which limits our ability to fully characterize treatment selection and potential confounding by indication. Future multi-center studies with extended follow-up periods are necessary to enable such analyses and to determine whether the early improvements we observed translate into sustained benefits in quality of life, adherence to treatment, and overall morbidity and mortality.

## Conclusion

Our QI initiative underscores the feasibility and clinical utility of integrating repeated PROMs into routine HD care, which appeared to improve patient–nurse agreement and perceived adequacy of assessment. The structured evaluation and adjustment phases enabled continuous, tailored interventions that were associated with improvements in the management of CKD-aP and depressive symptoms. By closing the gap between subjective perceptions and objective symptom measures, this approach has the potential to improve symptom-related patient outcomes and to align clinical practice with contemporary guidelines for holistic, patient-centered care.

## Supplementary Information

Below is the link to the electronic supplementary material.

Supplementary Material 1

## Data Availability

The data from the study will be shared upon request.
