# Supplementary material for: Reduction of pruritus and depression using longitudinal patient-reported outcome measures in hemodialysis: a quality improvement project
Source: J Patient Rep Outcomes. 2026 Jan 21;10:11. doi: 10.1186/s41687-026-01003-6 (PMC12827871; doi:10.1186/s41687-026-01003-6)
Supplement: Supplementary file 1 — Supplementary Material 1 [file 41687_2026_1003_MOESM1_ESM.docx]

**Reduction of pruritus and depression using longitudinal patient-reported outcome measures in hemodialysis**

**Table of contents:**

p. 2 Table S1: Questionnaire for patient at *Check-In*

p. 4 Table S2: Questionnaire for nurses at *Check-In*

p. 6 Table S3: Questionnaire for patient-reported outcome measures

p. 17 Table S4: Questionnaire for patient at *Check-Out*

p. 19 Table S5: Questionnaire for nurses at *Check-Out*

**Supplemental Tables**

**Table S1: Questionnaire for patients at *Check-In***

| **Item** | **Question** | **Choices** |
| --- | --- | --- |
| CHIP_01 | **Single Choice**  GER: Leiden Sie an Juckreiz?  *ENG: Do you suffer from itching?* | GER: Ja  *ENG: Yes* |
|  |  | GER: Nein  *ENG: No* |
| CHIP_02 | **Multiple Choice**  GER: Wo verspüren Sie Juckreiz?  *ENG: Where do you perceive itch?* | GER: Nirgends  *ENG: Nowhere* |
|  |  | GER: Beine  *ENG: Legs* |
|  |  | GER: Arme  *ENG: Arms* |
|  |  | GER: Rücken  *ENG: Back* |
|  |  | GER: Brust-& Bauchbereich  *ENG: Chest and abdomen* |
|  |  | GER: Kopf/Nackenbereich  *ENG: Head or neck* |
|  |  | GER: Hand- & Fußflächen  *ENG: Palms and foot soles* |
|  |  | GER: Am ganzen Körper  *ENG: Across the entire body* |
| CHIP_03 | **Single Choice**  GER: Wie würden Sie Ihren Flüssigkeitsstatus derzeit beschreiben?  *ENG: How would you categorize your fluid status?* | GER: Zu trocken  *ENG: Too dry* |
|  |  | GER: Genau richtig  *ENG: Exactly right* |
|  |  | GER: Überwässert  *ENG: Fluid overloaded* |
| CHIP_04 | **Single Choice**  GER: Leiden Sie an einer durch einen Arzt/eine Ärztin festgestellten Depression?  *ENG: Do you suffer from depression that was diagnosed by a doctor?* | GER: Ja  *ENG: Yes* |
|  |  | GER: Nein  *ENG: No* |
| CHIP_05 | **Single Choice**  GER: Finden Sie, dass Juckreiz derzeit adäquat erhoben wird?  *ENG: Do you think that itch is currently being adequately assessed?* | GER: Ja  *ENG: Yes* |
|  |  | GER: Nein  *ENG: No* |
| CHIP_06 | **Single Choice**  GER: Finden Sie, dass Ihr Flüssigkeitsstatus derzeit adäquat erhoben wird?  *ENG: Do you think that your fluid status is currently being adequately assessed?* | GER: Ja  *ENG: Yes* |
|  |  | GER: Nein  *ENG: No* |
| CHIP_07 | **Single Choice**  GER: Finden Sie, dass depressive Symptome derzeit adäquat erhoben werden?  *ENG: Do you think that symptoms of depression are currently being adequately assessed?* | GER: Ja  *ENG: Yes* |
|  |  | GER: Nein  *ENG: No* |
| CHIP_08 | **Single Choice**  GER: Produzieren Sie noch Restharn?  *ENG: Do you have residual diuresis?* | GER: Ja  *ENG: Yes* |
|  |  | GER: Nein  *ENG: No* |
| CHIP_09 | **Numerical Input**  GER: Wieviel Restharn in Milliliter (mL) haben Sie pro Tag?  *ENG: How much residual diuresis in millilitres (mL) do you have per day?* |  |
| CHIP_10 | **Single Choice**  GER: Erwarten Sie, dass Sie von diesem Projekt profitieren werden?  *ENG: Do you expect to benefit from this project?* | GER: Ja  *ENG: Yes* |
|  |  | GER: Nein  *ENG: No* |

**Table S2: Questionnaire for nurses at *Check-In***

| **Item** | **Question** | **Choices** |
| --- | --- | --- |
| CHIN_01 | **Single Choice**  GER: Leidet der Patient / die Patientin an Juckreiz?  *ENG: Does this patient suffer from itching* | GER: Ja  *ENG: Yes* |
|  |  | GER: Nein  *ENG: No* |
| CHIN_02 | **Single Choice**  GER: Hat der Patient / die Patientin sichtbare Kratzspuren?  *ENG: Does the patient have visible scratches?* | GER: Ja  *ENG: Yes* |
|  |  | GER: Nein  *ENG: No* |
| CHIN_03 | **Multiple Choice**  GER: Bekommt der Patient / die Patientin derzeit eine medikamentöse Therapie gegen Juckreiz?  *ENG: Does the patient currently receive medication for itching?* | GER: Nein  *ENG: No* |
|  |  | GER: Kapruvia®  *ENG: Kapruvia®* |
|  |  | GER: Gabapentin  *ENG: Gabapentin* |
|  |  | GER: Pregabalin  *ENG: Pregabalin* |
|  |  | GER: Cortison-haltige Crème  *ENG: Cream containing cortisone* |
|  |  | GER: Antihistaminika  *ENG: Antihistamines* |
|  |  | GER: Anderes  *ENG: Other* |
| CHIN_04 | **Single Choice**  GER: Wie würden Sie den Flüssigkeitsstatus des Patienten / der Patientin derzeit einschätzen?  *ENG: How would you categorize the patient’s fluid status?* | GER: Zu trocken  *ENG: Too dry* |
|  |  | GER: Genau richtig  *ENG: Exactly right* |
|  |  | GER: Überwässert  *ENG: Fluid overloaded* |
| CHIN_05 | **Single Choice**  GER: Leidet der Patient / die Patientin an einer durch einen Arzt / eine Ärztin festgestellten Depression?  *ENG: Does the patient suffer from depression that was diagnosed by a doctor?* | GER: Ja  *ENG: Yes* |
|  |  | GER: Nein  *ENG: No* |
|  | | |
| CHIN_06 | **Single Choice**  GER: Finden Sie, dass Juckreiz derzeit adäquat erhoben wird?  *ENG: Do you think that itch is currently being adequately assessed?* | GER: Ja  *ENG: Yes* |
|  |  | GER: Nein  *ENG: No* |
| CHIN_07 | **Single Choice**  GER: Finden Sie, dass Ihr Flüssigkeitsstatus derzeit adäquat erhoben wird?  *ENG: Do you think that your fluid status is currently being adequately assessed?* | GER: Ja  *ENG: Yes* |
|  |  | GER: Nein  *ENG: No* |
| CHIN_08 | **Single Choice**  GER: Finden Sie, dass depressive Symptome derzeit adäquat erhoben werden?  *ENG: Do you think that symptoms of depression are currently being adequately assessed?* | GER: Ja  *ENG: Yes* |
|  |  | GER: Nein  *ENG: No* |
| CHIN_09 | **Single Choice**  GER: Erwarten Sie, dass der Patient / die Patientin von diesem Projekt profitieren wird?  *ENG: Do you expect the patient to benefit from this project?* | GER: Ja  *ENG: Yes* |
|  |  | GER: Nein  *ENG: No* |
| CHIN_10 | **Single Choice**  GER: Erwarten Sie, dass das Pflege-Team von diesem Projekt profitieren wird?  *ENG: Do you exepect the nursing team to benefit from this project?* | GER: Ja  *ENG: Yes* |
|  |  | GER: Nein  *ENG: No* |
| CHIN_11 | **Single Choice**  GER: Erwarten Sie, dass das ärztliche Team von diesem Projekt profitieren wird?  *ENG: Do you expect the doctors to benefit from this project?* | GER: Ja  *ENG: Yes* |
|  |  | GER: Nein  *ENG: No* |

**Table S3: Questionnaire for patient-reported outcome measures**

| **Item** | **Question** | **Choices** |
| --- | --- | --- |
| SYMP_01 | GER: In welche dieser drei Kategorien ordnet sich der Patient / die Patientin innerhalb der letzten 14 Tage am ehesten ein?  *ENG: In which of these three categories does the patient most likely categorize himself/herself within the last 14 days?* |  |
| SYMP_02 | **Single Choice**  GER: Beispiel: Wenn der Patient / die Patientin angibt, sich regelmäßig "blutig" zu kratzen, aber keine Schlafprobleme angibt, ist er / sie in Kategorie 3 einzuordnen.  *ENG: Example: If the patient states that he/she regularly scratches him/herself ‘bloody’, but does not report any sleep problems, he/she should be categorized in category 3.* | GER: 1 - Mild: Ich habe normalerweise keine Kratzspuren auf meiner Haut. Ich habe normalerweise keine Schlafprobleme aufgrund von Juckreiz. Juckreiz löst in mir weder Wut noch Traurigkeit aus.  *ENG: 1 - Mild: I do not generally have scratch marks on my skin. I do not generally have a problem sleeping because of itching.  My itching does not generally make me feel agitated or sad.* |
|  |  | GER: 2 - Moderat: Ich habe manchmal Kratzspuren auf der Haut. Ich habe manchmal Schlafprobleme aufgrund von Juckreiz. Juckreiz löst in mir manchmal Wut oder Traurigkeit aus.  *ENG: 2 - Moderate: I sometimes have scratch marks on my skin.  I sometimes have problems sleeping because of itching. My itching can sometimes make me feel agitated or sad.* |
|  |  | GER: 3 - Schwer: Ich habe oft Kratzspuren auf der Haut, die bluten oder sich infizieren können, aber nicht müssen. Ich habe oft Schlafprobleme aufgrund von Juckreiz. Juckreiz löst in mir oft Wut oder Traurigkeit aus.  *ENG: 3 - Severe: I often have scratch marks on my skin that may or may not bleed or get infected. I often have a problem sleeping because of itching. My itching often makes me feel agitated or sad.* |
|  | | |
| SYMP_03 | **Multiple Choice**  GER: Bekommt der Patient / die Patientin derzeit eine medikamentöse Therapie gegen Juckreiz?  *ENG: Is the patient currently receiving medication to treat itching?* | GER: Nein.  *ENG: No.* |
|  |  | GER: Kapruvia®  *ENG: Kapruvia®* |
|  |  | GER: Gabapentin  *ENG: Gabapentin* |
|  |  | GER: Pregabalin  *ENG: Pregabalin* |
|  |  | GER: Cortison-haltige Crème  *ENG: Cream containing cortisone* |
|  |  | GER: Antihistaminika  *ENG: Antihistamines* |
|  |  | GER: Anderes  *ENG: Other* |
| SYMP_04 | GER: Wie würden Sie den Flüssigkeitsstatus des Patienten / der Patientin auf Basis folgender Fragen einschätzen?  *ENG: How would you assess the patient's fluid status based on the following questions?* |  |
| SYMP_05 | **Single Choice**  GER: Wie stark war in den letzten 14 Tagen die prädialytisch ausgeprägteste Atemnot des Patienten / der Patientin?  *ENG: How severe has the patient's pre-dialysis breathlessness been in the last 14 days?* | GER: Keine  *ENG: None* |
|  |  | GER: Im Liegen  *ENG: Lying down* |
|  |  | GER: Im Liegen mit zwei Kissen unter dem Kopf  *ENG: Lying down with two pillows under your head* |
|  |  | GER: Im Sitzen  *ENG: Sitting* |
| SYMP_06 | **Single Choice**  GER: Wie stark waren in den letzten 14 Tagen die prädialytisch ausgeprägtesten Knöchelödeme des Patienten / der Patientin?  *ENG: How severe have the patient's pre-dialysis ankle oedema been in the last 14 days?* | GER: Keine  *ENG: None* |
|  |  | GER: Schwachausgeprägt  *ENG: Moderately severe* |
|  |  | GER: Stark ausgeprägt  *ENG: Severe* |
|  | | |
| SYMP_07 | **Single Choice**  GER: Traten in den letzten 14 Tagen folgende Zeichen der Volumenexpansion zwischen Dialysen auf?  *ENG: Have the following signs of volume expansion occurred in the last 14 days between dialyses?* | GER: Keine  *ENG: None* |
|  |  | GER: Unerwartet geringe Zunahme der Körpermasse  *ENG: Unexpectedly small increase in body mass* |
|  |  | GER: Chronischer, neu aufgetretener Husten  *ENG: Chronic, new onset cough* |
| SYMP_08 | **Single Choice**  GER: Stieg der Blutdruck in den letzten 14 Tagen während der Dialyse eher an?  *ENG: Did blood pressure tend to increase during dialysis in the last 14 days?* | GER: Nein  *ENG: No* |
|  |  | GER: Ja  *ENG: Yes* |
| SYMP_09 | **Single Choice**  GER: Wie stark waren in den letzten 14 Tagen die postdialytisch ausgeprägtesten Wadenkrämpfe des Patienten / der Patientin?  *ENG: In the last 14 days, how severe were the patient's most pronounced postdialytic calf cramps of the patient?* | GER: Keine  *ENG: None* |
|  |  | GER: Schwach ausgeprägt  *ENG: Weak* |
|  |  | GER: Stark ausgeprägt  *ENG: Severe* |
| SYMP_10 | **Single Choice**  GER: Kam es in den letzten 14 lagen zu symptomatischen intradialytischen Hypotensionen und einem Abfall des systolischen Blutdrucks um ≥ 20 mmHg? Wie wurde interveniert?  *ENG: Did symptomatic intradialytic hypotension and a drop in systolic blood pressure of ≥ 20 mmHg occur in the last 14 days? What was the reaction?* | GER: Keine  *ENG: None* |
|  |  | GER: Wechsel der Körperposition  *ENG: Change of body position necessary* |
|  |  | GER: Kochsalzinfusion oder Stopp der Ultrafiltration nötig  *ENG: Saline infusion or stop ultrafiltration necessary* |
|  |  | GER: Erbrechen oder Bewusstlosigkeit als Folge  *ENG: Vomiting or unconsciousness as a result* |
| SYMP_11 | **Single Choice**  GER: Wie stark war in den letzten 14 Tagen das am stärksten ausgeprägteste Zeichen von Volumendepletion, das Sie bei diesem Patienten mitbekommen haben?  *ENG: How strong was the most pronounced sign of volume depletion that you have seen in this patient in the last 14 days?* | GER: Keine  *ENG: None* |
|  |  | GER: Durst direkt nach der Dialyse *ENG: Thirst right after dialysis* |
|  |  | GER: Schlaffheit oder Müdigkeit  *ENG: Sleepiness or tiredness* |
|  |  | GER: Schwindel oder symptomatische Hypotension  *ENG: Dizziness or symptomatic hypotension* |
| SYMP_12 | GER: Bitte fragen Sie den Patienten / die Patientin, wie oft er / sie sich im Verlauf der letzten 14 Tage durch die folgenden Beschwerden beeinträchtigt gefühlt hat:  *ENG: Please ask the patient how often he/she has felt affected by the following complaints in the last 14 days:* |  |
|  | | |
| SYMP_13 | **Single Choice**  GER: Wenig Interesse oder Freude an Ihren Tätigkeiten  *ENG:  Little interest or pleasure in doing things* | GER: Überhaupt nicht  *ENG: Not at all* |
|  |  | GER: An einzelnen Tagen  *ENG: Several days* |
|  |  | GER: An mehr als der Hälfte der Tage  *ENG: More than half the days* |
|  |  | GER: Beinahe jeden Tag  *ENG: Nearly every day* |
| SYMP_14 | **Single Choice**  GER: Niedergeschlagenheit, Schwermut oder Hoffnungslosigkeit  *ENG: Feeling down, depressed, or hopeless* | GER: Überhaupt nicht  *ENG: Not at all* |
|  |  | GER: An einzelnen Tagen  *ENG: Several days* |
|  |  | GER: An mehr als der Hälfte der Tage  *ENG: More than half the days* |
|  |  | GER: Beinahe jeden Tag  *ENG: Nearly every day* |
| SYMP_15 | **Single Choice**  GER: Schwierigkeiten ein- oder durchzuschlafen oder vermehrter Schlaf  *ENG:  Trouble falling or staying asleep, or sleeping too much* | GER: Überhaupt nicht  *ENG: Not at all* |
|  |  | GER: An einzelnen Tagen  *ENG: Several days* |
|  |  | GER: An mehr als der Hälfte der Tage  *ENG: More than half the days* |
|  |  | GER: Beinahe jeden Tag  *ENG: Nearly every day* |
| SYMP_16 | **Single Choice**  GER: Müdigkeit oder Gefühl, keine Energie zu haben  *ENG: Feeling tired or having little energy* | GER: Überhaupt nicht  *ENG: Not at all* |
|  |  | GER: An einzelnen Tagen  *ENG: Several days* |
|  |  | GER: An mehr als der Hälfte der Tage  *ENG: More than half the days* |
|  |  | GER: Beinahe jeden Tag  *ENG: Nearly every day* |
|  | | |
| SYMP_17 | **Single Choice**  GER: Verminderter Appetit oder übermäßiges Bedürfnis zu essen  *ENG:  Poor appetite or overeating* | GER: Überhaupt nicht  *ENG: Not at all* |
|  |  | GER: An einzelnen Tagen  *ENG: Several days* |
|  |  | GER: An mehr als der Hälfte der Tage  *ENG: More than half the days* |
|  |  | GER: Beinahe jeden Tag  *ENG: Nearly every day* |
| SYMP_18 | **Single Choice**  GER: Schlechte Meinung von sich selbst; Gefühl, ein Versager zu sein oder die Familie enttäuscht zu haben  *ENG: Feeling bad about yourself — or that you are a failure or*  *have let yourself or your family down* | GER: Überhaupt nicht  *ENG: Not at all* |
|  |  | GER: An einzelnen Tagen  *ENG: Several days* |
|  |  | GER: An mehr als der Hälfte der Tage  *ENG: More than half the days* |
|  |  | GER: Beinahe jeden Tag  *ENG: Nearly every day* |
| SYMP_19 | **Single Choice**  GER: Schwierigkeiten, sich auf etwas zu konzentrieren, z.B. beim Zeitunglesen oder Fernsehen  *ENG: Trouble concentrating on things, such as reading the*  *newspaper or watching television* | GER: Überhaupt nicht  *ENG: Not at all* |
|  |  | GER: An einzelnen Tagen  *ENG: Several days* |
|  |  | GER: An mehr als der Hälfte der Tage  *ENG: More than half the days* |
|  |  | GER: Beinahe jeden Tag  *ENG: Nearly every day* |
| SYMP_20 | **Single Choice**  GER: Waren Ihre Bewegungen oder Ihre Sprache so verlangsamt, dass es auch anderen auffallen würde? Oder waren Sie im Gegenteil „zappelig" oder ruhelos und hatten dadurch einen stärkeren Bewegungsdrang als sonst?  *ENG: Moving or speaking so slowly that other people could have noticed? Or the opposite — being so fidgety or restless that you have been moving around a lot more than usual* | GER: Überhaupt nicht  *ENG: Not at all* |
|  |  | GER: An einzelnen Tagen  *ENG: Several days* |
|  |  | GER: An mehr als der Hälfte der Tage  *ENG: More than half the days* |
|  |  | GER: Beinahe jeden Tag  *ENG: Nearly every day* |
|  | | |
| SYMP_21 | **Single Choice**  GER: Gedanken, dass Sie lieber tot wären oder sich Leid zufügen möchten  *ENG: Thoughts that you would be better off dead or of hurting*  *yourself in some way* | GER: Überhaupt nicht  *ENG: Not at all* |
|  |  | GER: An einzelnen Tagen  *ENG: Several days* |
|  |  | GER: An mehr als der Hälfte der Tage  *ENG: More than half the days* |
|  |  | GER: Beinahe jeden Tag  *ENG: Nearly every day* |

| **Item** | **Question** | **Choices** |
| --- | --- | --- |
| PROM_01 | **Single Choice**  *GER: Wie fühlten Sie sich heute direkt nach Ihrer Dialyse?*  *ENG: How did you feel right after dialysis today?* | GER: Sehr gut  *ENG:* Very good |
|  |  | GER: Good  *ENG: Good* |
|  |  | GER: Neutral  *ENG: Neutral* |
|  |  | GER: Eher schlecht  *ENG: Rather bad* |
|  |  | GER: Schlecht  *ENG: Bad* |
| PROM_02 | **Single Choice**  GER: Wählen Sie jene Zahl aus, welche die Qualität Ihres Schlafes in den letzten 24 Stunden am besten beschreibt!  *ENG: Choose the number best representing your sleep quality within the previous 24 hours!* | GER: 0 – Bestmöglicher Schlaf  *ENG: 0 – Best possible sleep* |
|  |  | 1 |
|  |  | 2 |
|  |  | 3 |
|  |  | 4 |
|  |  | 5 |
|  |  | 6 |
|  |  | 7 |
|  |  | 8 |
|  |  | 9 |
|  |  | GER: 10 – Schlechtester Schlaf  *ENG: 10* *– Worst sleep* |
|  | | |
| PROM_03 | **Single Choice**  GER: Bitte bewerten Sie den schlimmsten Juckreiz, den Sie den letzten 24 Stunden verspürt haben!  *ENG: Please rate the worst itch you have perceived during the previous 24 hours!* | GER: 0 – Kein Juckreiz  *ENG: 0 – No tich* |
|  |  | GER: 1 – Mild  *ENG: 1 – Mild* |
|  |  | GER: 2 – Mild  *ENG: 2 – Mild* |
|  |  | GER: 3 – Mild  *ENG: 3 – Mild* |
|  |  | GER: 4 – Moderat  *ENG: 4 – Moderat* |
|  |  | GER: 5 – Moderat  *ENG: 5 – Moderat* |
|  |  | GER: 6 – Moderat  *ENG: 6 – Moderat* |
|  |  | GER: 7 – Stark  *ENG: 7 – Severe* |
|  |  | GER: 8 – Stark  *ENG: 8 – Severe* |
|  |  | GER: 9 – Schlimmster vorstellbarer Juckreiz  *ENG: 9 – Worst imaginable itch* |
|  |  | GER: 10 – Schlimmster vorstellbarer Juckreiz  *ENG: 10 – Worst imaginable itch* |
| PROM_04 | **Single Choice**  GER: Wie lange hat es nach der letzten Dialyse gedauert, bis Sie sich erholt haben?  *ENG: When did you recover after the previous dialysis treatment?* | GER: Weniger als 2 Stunden  *ENG: Less than 2 hours* |
|  |  | GER: 2 bis 6 Stunden  *ENG: 2 to 6 hours* |
|  |  | GER: 7 bis 12 Stunden  *ENG: 7 to 12 hours* |
|  |  | GER: Mehr als 12 Stunden  *ENG: More than 12 hours* |
|  | | |
| PROM_05 | **Single Choice**  GER: Wie schätzen Sie Ihre heutige Dialyse ein?  *ENG: How would you rate today’s dialysis?* | GER: 😃 Sehr gut  *ENG:* 😃 Very good |
|  |  | GER: Good  *ENG: Good* |
|  |  | GER: Neutral  *ENG: Neutral* |
|  |  | GER: Eher schlecht  *ENG: Rather bad* |
|  |  | GER: Schlecht  *ENG: Bad* |
| PROM_06 | **Multiple Choice**  GER: Gab es besondere Vorkommnisse während der heutigen Dialyse?  *ENG: Did any complications occur during today’s dialysis?* | GER: Nein  *ENG: No* |
|  |  | GER: Spürbarer Abfall des Blutdrucks  *ENG: Perceivable drop in blood pressure* |
|  |  | GER: Spürbarer Anstieg des Blutdrucks  *ENG: Perceivable rise in blood pressure* |
|  |  | GER: Blutung  *ENG: Bleeding* |
|  |  | GER: Probleme mit dem Dialysezugang  *ENG: Dialysis access issues* |
|  |  | GER: Krämpfe  *ENG: Cramps* |
| PROM_07 | **Multiple Choice**  GER: Was wurde gegen den Blutdruckabfall unternommen? *ENG: Which measure was taken to mitigate the drop in blood pressure?* | GER: Nichts  *ENG: None* |
|  |  | GER: Änderung der Körperposition  *ENG: Change In body position* |
|  |  | GER: Infusion von Flüssigkeit  *ENG: Fluid infusion* |
| PROM_08 | **Numerical Input**  GER: Wie hoch war Ihr Körpergewicht nach der heutigen Dialyse?  *ENG: What was your body weight after dialysis today?* |  |
| PROM_09 | **Numerical Input**  GER: Wie hoch war Ihr heutiges Zielgewicht?  *ENG: What was your target weight today?* |  |
| PROM_10 | **Single Choice**  GER: Ziehen Sie von Ihrem Körpergewicht etwas für Ihre Kleidung ab?  *ENG: Do you deduct weight from your body weight to account for clothing?* | GER: Ja  *ENG: Yes* |
|  |  | GER: Nein  *ENG: No* |
| PROM_11 | **Multiple Choice**  GER: Bekommen Sie derzeit eine medikamentöse Therapie gegen Juckreiz? *ENG: Do you currently receive medication for itching?* | GER: Nein  *ENG: No* |
|  |  | GER: Kapruvia®  *ENG: Kapruvia®* |
|  |  | GER: Gabapentin  *ENG: Gabapentin* |
|  |  | GER: Pregabalin  *ENG: Pregabalin* |
|  |  | GER: Cortison-haltige Crème  *ENG: Cream containing cortisone* |
|  |  | GER: Antihistaminika  *ENG: Antihistamines* |
|  |  | GER: Anderes  *ENG: Other* |
| PROM_12 | **Multiple Choice**  GER: Wurde seit der letzten Dialyse eine Änderung Ihres Trockengewichts durchgeführt?  *ENG: Was your dry weight changed since the last dialysis treatment?* | GER: Nein  *ENG: No* |
|  |  | GER: Ja, von mir  *ENG: Yes, by myself* |
|  |  | GER: Ja, von der Pflegeperson  *ENG: Yes, by the nurse* |
|  |  | GER: Ja, von der Ärzteschaft  *ENG: Yes, by the doctors* |
|  | | |
| PROM_13 | **Multiple Choice**  GER: Wurde Ihnen seit der letzten Dialyse zu einer psychaitrischen Begutachtung geraten?  *ENG: Did you receive a recommendation to see a psychiatrist?* | GER: Nein  *ENG: No* |
|  |  | GER: Ja, von der Pflegeperson  *ENG: Yes, by the nurse* |
|  |  | GER: Ja, von der Ärzteschaft  *ENG: Yes, by the doctors* |

**Table S4: Questionnaire for patients at *Check-Out***

| **Item** | **Question** | **Choices** |
| --- | --- | --- |
| CHOP_01 | **Single Choice**  GER: Leiden Sie an Juckreiz?  *ENG: Do you suffer from itching?* | GER: Ja  *ENG: Yes* |
|  |  | GER: Nein  *ENG: No* |
| CHOP_02 | **Multiple Choice**  GER: Wo verspüren Sie Juckreiz?  *ENG: Where do you perceive itch?* | GER: Nirgends  *ENG: Nowhere* |
|  |  | GER: Beine  *ENG: Legs* |
|  |  | GER: Arme  *ENG: Arms* |
|  |  | GER: Rücken  *ENG: Back* |
|  |  | GER: Brust-& Bauchbereich  *ENG: Chest and abdomen* |
|  |  | GER: Kopf/Nackenbereich  *ENG: Head or neck* |
|  |  | GER: Hand- & Fußflächen  *ENG: Palms and foot soles* |
|  |  | GER: Am ganzen Körper  *ENG: Across the entire body* |
| CHOP_03 | **Single Choice**  GER: Wie würden Sie Ihren Flüssigkeitsstatus derzeit beschreiben?  *ENG: How would you categorize your fluid status?* | GER: Zu trocken  *ENG: Too dry* |
|  |  | GER: Genau richtig  *ENG: Exactly right* |
|  |  | GER: Überwässert  *ENG: Fluid overloaded* |
| CHOP_04 | **Single Choice**  GER: Leiden Sie an einer durch einen Arzt/eine Ärztin festgestellten Depression?  *ENG: Do you suffer from depression that was diagnosed by a doctor?* | GER: Ja  *ENG: Yes* |
|  |  | GER: Nein  *ENG: No* |
|  | | |
| CHOP_05 | **Single Choice**  GER: Finden Sie, dass im Rahmen des Projekts Juckreiz adäquat erhoben wurde?  *ENG: Do you think that itch was adequately assessed during the project?* | GER: Ja  *ENG: Yes* |
|  |  | GER: Nein  *ENG: No* |
| CHOP_06 | **Single Choice**  GER: War Ihnen der Aufwand zur Juckreiz-Erhebung zu hoch?  *ENG: Did itch assessment require too much effort?* | GER: Ja  *ENG: Yes* |
|  |  | GER: Nein  *ENG: No* |
| CHOP_07 | **Single Choice**  GER: Finden Sie, dass im Rahmen des Projekts Ihr Flüssigkeitsstatus erhoben wurde?  *ENG: Do you think that your fluid status was adequately assessed during the project?* | GER: Ja  *ENG: Yes* |
|  |  | GER: Nein  *ENG: No* |
| CHOP_08 | **Single Choice**  GER: War Ihnen der Aufwand zur Flüssigkeits-Erhebung zu hoch?  *ENG: Did fluid status assessment require too much effort?* | GER: Ja  *ENG: Yes* |
|  |  | GER: Nein  *ENG: No* |
| CHOP_09 | **Single Choice**  GER: Finden Sie, dass im Rahmen des Projekts depressive Symptome adäquat erhoben wurden?  *ENG: Do you think that symptoms of depression were adequately assessed during the project?* | GER: Ja  *ENG: Yes* |
|  |  | GER: Nein  *ENG: No* |
| CHOP_10 | **Single Choice**  GER: War Ihnen der Aufwand zur Depressions-Erhebung zu hoch?  *ENG: Did depression assessment require too much effort?* | GER: Ja  *ENG: Yes* |
|  |  | GER: Nein  *ENG: No* |
| CHOP_11 | **Single Choice**  GER: Finden Sie, dass Sie von diesem Projekt insgesamt profitiert haben?  *ENG: Do you believe to have benefited from this project overall?* | GER: Ja  *ENG: Yes* |
|  |  | GER: Nein  *ENG: No* |

**Table S5: Questionnaire for nurses at *Check-Out***

| **Item** | **Question** | **Choices** |
| --- | --- | --- |
| CHON_01 | **Single Choice**  GER: Leidet der Patient / die Patientin an Juckreiz?  *ENG: Does this patient suffer from itching* | GER: Ja  *ENG: Yes* |
|  |  | GER: Nein  *ENG: No* |
| CHON_02 | **Single Choice**  GER: Hat der Patient / die Patientin sichtbare Kratzspuren?  *ENG: Does the patient have visible scratches?* | GER: Ja  *ENG: Yes* |
|  |  | GER: Nein  *ENG: No* |
| CHON_03 | **Multiple Choice**  GER: Bekommt der Patient / die Patientin derzeit eine medikamentöse Therapie gegen Juckreiz?  *ENG: Does the patient currently receive medication for itching?* | GER: Nein  *ENG: No* |
|  |  | GER: Kapruvia®  *ENG: Kapruvia®* |
|  |  | GER: Gabapentin  *ENG: Gabapentin* |
|  |  | GER: Pregabalin  *ENG: Pregabalin* |
|  |  | GER: Cortison-haltige Crème  *ENG: Cream containing cortisone* |
|  |  | GER: Antihistaminika  *ENG: Antihistamines* |
|  |  | GER: Anderes  *ENG: Other* |
| CHON_04 | **Single Choice**  GER: Wie würden Sie den Flüssigkeitsstatus des Patienten / der Patientin derzeit einschätzen?  *ENG: How would you categorize the patient’s fluid status?* | GER: Zu trocken  *ENG: Too dry* |
|  |  | GER: Genau richtig  *ENG: Exactly right* |
|  |  | GER: Überwässert  *ENG: Fluid overloaded* |
| CHON_05 | **Single Choice**  GER: Leidet der Patient / die Patientin an einer durch einen Arzt / eine Ärztin festgestellten Depression?  *ENG: Does the patient suffer from depression that was diagnosed by a doctor?* | GER: Ja  *ENG: Yes* |
|  |  | GER: Nein  *ENG: No* |
|  | | |
| CHON_06 | **Single Choice**  GER: Finden Sie, dass im Rahmen des Projekts bei diesem Patienten / dieser Patientin Juckreiz adäquat erhoben wurde?  *ENG: Do you think that itch was adequately assessed in this patient during the project?* | GER: Ja  *ENG: Yes* |
|  |  | GER: Nein  *ENG: No* |
| CHON_07 | **Single Choice**  GER: Finden Sie, dass im Rahmen des Projekts bei diesem Patienten / dieser Patientin der Flüssigkeitsstatus adäquat erhoben wurde?  *ENG: Do you think that the fluid status was adequately assessed in this patient during the project?* | GER: Ja  *ENG: Yes* |
|  |  | GER: Nein  *ENG: No* |
| CHON_08 | **Single Choice**  GER: Finden Sie, dass im Rahmen des Projekts bei diesem Patienten / dieser Patientin Depression adäquat erhoben wurde?  *ENG: Do you think depression was adequately assessed in this patient during the project?* | GER: Ja  *ENG: Yes* |
|  |  | GER: Nein  *ENG: No* |
